# A Bill to Regulate the Employment of, and Pay for, Medical Expert Testimony

**Published:** 1897-07

**Authors:** Henry W. Terrell

**Affiliations:** Greenville, Ga.


### A BILL TO REGULATE THE EMPLOYMENT OF, AND PAY FOR, MEDICAL EXPERT TESTIMONY.

GREENVILLE, GA., June 12, 1897.

*Editor Atlanta Medical and Surgical Journal:*

Being professionally hindered, it was not my pleasure to attend the recent meeting of the Georgia Medical Association, held at Macon. However, I saw from the daily papers that a committee was appointed by the President to have presented before the Georgia legislature a bill in regard to compensation for medical expert testimony. At the last session of the legislature I framed a bill of this character, and had one of our representatives, Dr. Jas.

W. Taylor, to introduce it. The bill is still before the Judiciary Committee of the house. Not knowing the personnel of the committee appointed by the Association (not having been present at the meeting), I take this method of giving the above information. Should they see fit to read the bill, they can obtain it through the clerk of the House of Representatives.

Hoping this bit of information may be of some service to the committee, I am, yours truly,

HENRY W. TERRELL, M.D.

[Following is this bill:

A bill to be entitled an act to regulate the employment and pay for medical expert testimony.

Section 1. Be it enacted by the General Assembly of Georgia, and it is hereby enacted by authority of the same, That whenever any person is confined in prison under indictment for murder, attempt to murder, manslaughter, arson, highway robbery, forgery, or any felony, and medical expert testimony is required and so demanded by the prisoner in his or her defense, whether of a surgical, medical or clinical nature, he or his attorney shall so inform the presiding judge at or before his arraignment for trial, whereupon the presiding judge before whom such trial is pending shall appoint such expert or experts as may be necessary to adequately represent both State and defense, and the compensation for such expert service shall be fixed by an order of the court at a rate that shall be reasonable for professional services of such a nature.

Sec. 2. Be it further enacted by the authority aforesaid, That the expert or experts so appointed shall be of repute, and a graduate of some recognized medical college and qualified in the branch of medical science to which the question calling for expert opinion relates, and shall have full and free access to the evidence adduced on trial, as well as to the defendant, if the issue involves his mental or physical state. On the completion of the examination, the said expert or experts shall submit to the court and jury, either in writing or verbal from the stand, under oath, their opinion, setting forth the reasons for said opinion, and facts upon which said opinion is based. If counsel on either side shall demand it, the expert shall be sworn as a witness, but their questions, both direct and cross, shall be limited to the facts and opinions as are given in the expert's statement.

Sec. 3. Be it further enacted by the authority aforesaid, That all laws or parts of laws inconsistent with this Act be, and the same are, hereby repealed.—*Ed.*]

